# Risk Factors and Spatial Distribution of *Schistosoma mansoni* Infection among Preschool-Aged Children in Blapleu, Biankouma District, Western Côte d'Ivoire

**DOI:** 10.1155/2021/6224401

**Published:** 2021-11-28

**Authors:** Constant Konan N'Zi, Mamadou Ouattara, Rufin Kouassi Assaré, Fidèle Kouakou Bassa, Nana Rose Diakité, Eliézer Kouakou N'Goran

**Affiliations:** ^1^Institut National de Santé Publique, Abidjan BP V 46, Côte d'Ivoire; ^2^Université Félix Houphouët-Boigny, 22 BP 582, Abidjan 22, Côte d'Ivoire; ^3^Centre Suisse de Recherches Scientifiques en Côte D'Ivoire, 01 BP 1303, Abidjan, Côte d'Ivoire; ^4^Swiss Tropical and Public Health Institute, CH-4002, Basel, Switzerland; ^5^University of Basel, CH-4003, Basel, Switzerland

## Abstract

*Schistosoma mansoni* infection is common among school-age children (SAC) in western Côte d'Ivoire. Little is known on the infection rate of preschool-aged children (PSAC) due to epidemiological data deficiency and nonappropriate formulation of the drug. Thus, mass drug administration for schistosomiasis control mainly targets SAC. This study aims to identify the risk factors and spatial distribution of *S. mansoni* infection among PSAC in Blapleu, endemic foci of *S. mansoni*. We carried out a cross-sectional study in households with PSAC aged 1–6 years. A structured questionnaire was administered to mothers/guardians to obtain data on sociodemographics and water contact behaviour of children. Point-of-care circulating cathodic antigen (POC-CCA) immunodiagnostic test in urine and Kato-Katz (K-K) method with stool were used for *S. mansoni* infection diagnosis. Multiple logistic regression analysis was performed to determine the relationship between *S. mansoni* infection and sociodemographic data. Coordinates recorded by a Global Positioning System of households, water source points, and infected PSAC were used to map the spatial distribution of *S. mansoni* infection cases. This study was conducted with 350 PSAC aged 1–6 years. The overall infection prevalence of *S. mansoni* varies from 31.43% with the K-K method to 62.86% with the POC-CCA. PSAC aged 2–6 years were highly infected with *S. mansoni* than those aged 1-2 years (OR = 14.24, 95% CI: 5.85–34.64). PSAC who did not have access and who do not live close to the infected water source were at a significant lower risk of *S. mansoni* infection (OR = 0.13, 95% CI: 0.057–0.30). The main purpose of water contact of PSAC was to help their mother for laundry that occurs weekly. In Blapleu, a high risk of *S. mansoni* infection was observed among PSAC. Schistosomiasis control effort in such localities should include information, education, and communication, water, sanitation, and hygiene, and particularly chemotherapy targeting PSAC, reinforcing the need of the paediatric praziquantel formulation.

## 1. Introduction

Schistosomiasis is a neglected tropical disease (NTD) caused by worms (trematodes) of the genus *Schistosoma*. There are two main forms of schistosomiasis, namely, intestinal schistosomiasis and urogenital schistosomiasis. In 2018, at least 290.8 million people worldwide required preventive treatment for schistosomiasis, and more than 97.2 million people received the treatment [1]. In children, schistosomiasis is associated with chronic anaemia, nutritional impairment, stunted growth, and mental slowness [2, 3]. Schistosomiasis can lead to cognitive impairment and impacts negatively on children's educational outcomes [4, 5]. Schistosomiasis is still a major public health challenge in sub-Saharan Africa [6].

In Côte d'Ivoire, western region is a schistosomiasis hyperendemic area with prevalence ranging from 50% to 100% in several settings [7–9]. Thus, the country adopted the WHO's approach of including mass drug administration (MDA) in NTD control programmes. Accordingly, MDA with praziquantel targeting 6- to 15-year children is implemented since 2011 in endemic areas. This approach fails to include preschool-aged children (PSAC) due to a lack of paediatric praziquantel treatment and epidemiological data [10–13]. Some previous studies have shown in PSAC high prevalence and serious burden on *S. mansoni* infection [14–17]. Therefore, there is a need to include PSAC in preventive chemotherapy for better schistosomiasis control [18–20].

Since 2016, the paediatric praziquantel consortium composed of Japan, Germany, Switzerland, the Netherlands, Brazil, the United Kingdom, Kenya, and Côte d'Ivoire is conducting a clinical trial aimed to find a paediatric praziquantel drug formulation. The first part of this Phase II clinical trial took place in western Côte d'Ivoire. Blapleu city has been particularly affected by schistosomiasis among PSAC aged 2 to 6 years (unpublished data). This study aims to improve our knowledge about the disease transmission, the risk factors, and the spatial distribution of *S. mansoni* infection in PSAC living in Blapleu.

## 2. Materials and Methods

### 2.1. Ethical Consideration

Ethical clearance was obtained from Comité National d'Ethique et de la Recherche of the Ministry of Public Health and Hygiene of Côte d'Ivoire (reference no. 046/MSHP/CNER-Kp). Written informed consent was systematic for any child's enrolment in the study. The parent/legal guardian could withdraw his child from the study at any time. PSAC infected with *S. mansoni* were offered free medical checkups and paediatric praziquantel treatment (currently being tested); any other diseases diagnosed on further investigation were managed. Data collected from each child were strictly confidential.

### 2.2. Study Area

**Figure 1:**
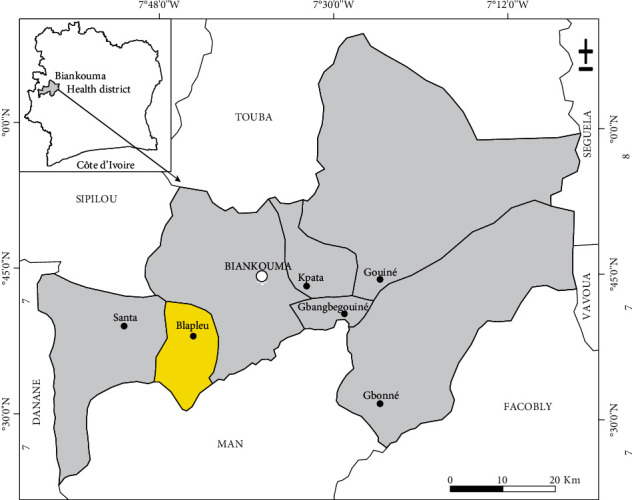
Map of western Côte d'Ivoire indicating the study area (in yellow color).

The study was carried out in Blapleu (latitude: 7° 37′ 59.9874″; longitude: -7° 43′ 59.9874″), located in Biankouma health district, Tonkpi region, western Côte d'Ivoire (Figure 1). Tonkpi region is a mountainous area with an average elevation ranging from 300 m above mean sea level (alms) to slightly above 1000 m amsl [7]. The climate is subtropical with average temperatures ranging up to 24°C; the rainy season extends from March to October [21]. According to the Institut National de la Statistique de Côte d'Ivoire, there were 14750 inhabitants [22], mostly natives belonging to the “Yacouba” ethnic group. Drinking water is obtained from household wells and pumps. There is a permanent river as well as two primary schools and one health center with trained medical practitioners.

### 2.3. Study Design, Periods, and Inclusion Criteria

A cross-sectional study was conducted from June 2016 to December 2017 for PSAC aged 2–6 years and from July to September 2018 for PSAC aged 1-<2 years. Children who lived in Blapleu and did not receive any antimalarial or anthelmintic treatment in the last four weeks were eligible.

### 2.4. Sociodemographic Data Collection

A questionnaire was tested and administered to PSAC parents/legal guardians to collect data on sociodemographics and human-water contact behaviours. First, the households were visited by three chosen community health workers for the identification of PSAC aged 1-2 years. Second, parents/legal guardians with previously identified children were invited to meet the researcher at the Blapleu health center within an agreed time schedule. Children with birth certificate, with written informed consent signed by their parents/legal guardians, and who fulfilled all the aforementioned eligibility criteria were enrolled in the study. Third, the interviewer went to all households in order to administer the questionnaire and to visit with parents'/legal guardian's water point of each selected household. Geographical coordinates were collected by using a GPS (eTrex H, Deutschland, Garmin International, Germany) at the center of each household and at a distance of one meter away from each visited water source.

### 2.5. Parasitological Survey

A paediatric urinal collector was set up for each PSAC to sample about 10 ml of urine. Urine samples were subjected to point-of-care (POC) circulating cathodic antigen (CCA) test (Schisto POC-CCA cassette-based test, Rapid Medical Diagnostics, Pretoria, South Africa). In brief, two drops of urine were transferred to the well of the cassette. After 20 minutes of absorbance time, the tests were read by an experienced technician. The results were scored as negative or positive (trace results were considered as positive).

Parents of POC-CCA-positive children were given two plastic containers and asked to collect two stool samples of their PSAC at home. The first stool sample was collected, and the second stool sample was provided within sixth days after the first. Stool samples were transferred to the Centre Hospitalier Regional (CHR) de Man and proceeded for *S. mansoni* eggs' examination according to the Kato-Katz method [23]. Triplicate 47.1 mg Kato-Katz thick smears were prepared per stool sample. After 60 minutes of clearing times, the thick smears were read by three experienced parasitologists. The number of helminth eggs of *S. mansoni* and soil-transmitted helminth was counted and recorded. Positive results were confirmed if at least two *S. mansoni* eggs were observed from a slide. For quality control, 10% of the slide were reexamined by one of the senior technicians.

### 2.6. Data Statistical Analysis

Data analysis was performed using STATA (version IC13.1; Stata Corporation, College Station, TX). The relationship between *S. mansoni* infection and the potential risk factors was evaluated by a multivariate logistic regression model. ArcMap (version 10.5.1, Environmental Systems Research Institute Inc.; Redlands, California, United States of America) was used to determine distances between households and the water source points. ArcView (version 3.2, Redlands, USA) was used to generate the map of households, water source points, and spatial distribution of *S. mansoni* infection cases.

## 3. Results

### 3.1. Sociodemographic Characteristics of Study Participants

Overall, 350 PSAC including 193 males (55.14%) and 157 females (44.86%) participated in the study (Table 1). There were less children aged 1-<2 years, 105 (30%), than the ones aged 2–6 years, 245 (70%). Most of the children mainly lived in traditional households (90.29%) with 1 to 3 rooms (74.57%) and 3 to 6 inhabitants (67.15%). Latrine availability was limited (19.86%), and household water was provided mainly from traditional boreholes (83.75%). Inhabitants of over half of the households were foreigners (56%). Islam was the religion most practiced (43.43%), and most of the mothers were illiterate (76.57%).

### 3.2. Prevalence of *S. mansoni* Infection Based on the POC-CCA and K-K Methods

Based on the POC-CCA method, 62.86% (220/350) children were infected with *S. mansoni*. There was no difference in the *S. mansoni* prevalence in PSAC between male and female and age groups (Table 2).

Using K-K, the overall *S. mansoni* prevalence was 31.43% (110/350). The *S. mansoni* prevalence infection was significantly higher in children aged 2–6 years (40.41%, 95% CI: 57.47–72.79%) compared with those aged 1-<2 years (10.48%, 95% CI: 7.20–25.15%), and there was no significant gender difference (Table 2).

### 3.3. Frequency of Water Source Point Visits

Nearly half of PSAC (49.14%) were regularly visiting a water source point together with their mothers/legal guardians (89.39%). Mothers/legal guardians were visiting water source points at least weekly (78.95%) and washing clothes accounted with 83.83%. Most PSAC (86.24%) were exposed to water source points through swimming and laundry, but 25% of PSAC, namely, those aged 1-<2 years (*n* = 13), were carried on their mother's backs with no visible water contact. About 20% of PSAC aged 2–6 years go alone to water source points for recreation activities (Table 3).

### 3.4. Risk Factors Associated with *S. mansoni* Infection in PSAC

**Figure 2:**
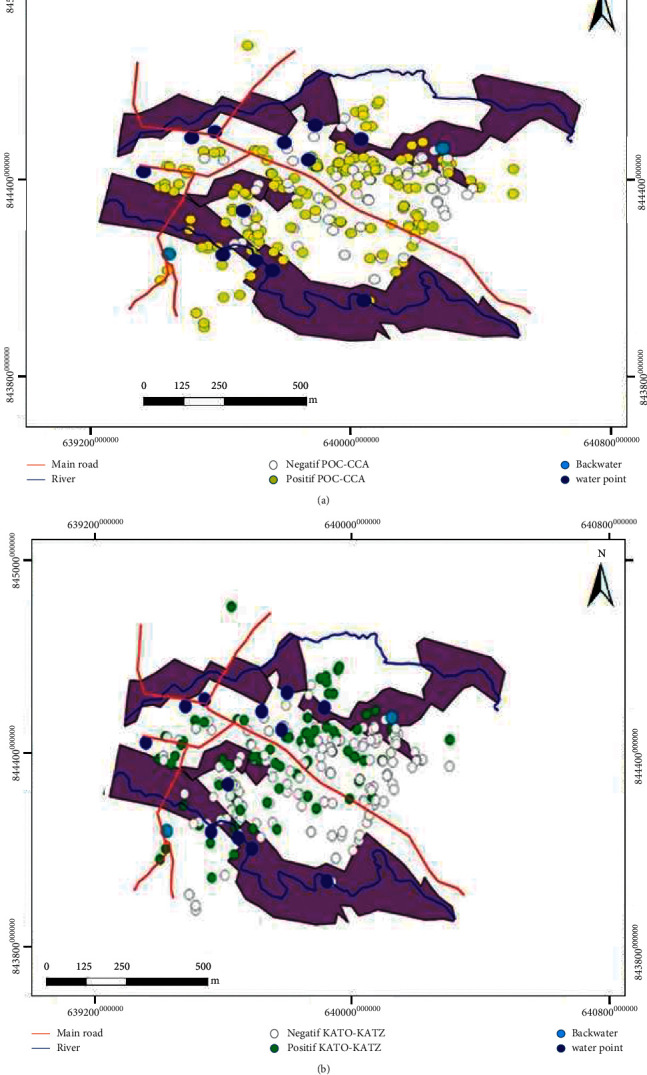
*Schistosoma mansoni* infections based on POC-CCA (a) and K-K (b) methods among PSAC in Blapleu, Western Côte d'Ivoire.

Multivariate logistic regression analysis on sociodemographic data, water contact pattern, and distance from the household to water source points has identified two main risk factors. Children aged 2–6 years had significantly higher odds of *S. mansoni* infection compared to 1- <2-year children (OR = 14.24, 95% CI: 5.85–34.64), while children not exposed to the water point had lower odds of *S. mansoni* infection (OR = 0.13, 95% CI: 0.057–0.30). Spatial distribution was homogeneous in *S. mansoni* infection using POC-CCA (Figure 2(a)). In contrast, K-K method showed that *S. mansoni* infections were distributed in a concentric pattern close to water source points (Figure 2(b)).

## 4. Discussion

This study provides knowledge and information on *S. mansoni* infection epidemiology of PSAC living in a highly endemic locality in western Côte d'Ivoire. The prevalence of *S. mansoni* infection, risk factors, and spatial distribution in this area were identified. The overall *S. mansoni* prevalence among PSAC observed from this study was very high compared to the reported data in Côte d'Ivoire [16]. Indeed, the western region is still considered to be a major endemic area for *S. mansoni* in Côte d'Ivoire [7, 8].

The prevalence of *S. mansoni* infection using the POC-CCA test was two times higher than the K-K method (62.86% versus 31.43%). Previous studies carried out in western Côte d'Ivoire [24] and other countries also confirm this result [17, 25, 26]. In addition, POC-CCA tests were reported to be 3 to 9 times more sensitive than the K-K method [27, 28]. POC-CCA sensitivity and specificity ranged from 76.7 to 99.1% and 75%, respectively [15, 29]. However, POC-CCA test findings are speculative due to the detection of antibodies to *S. mansoni* which could either confirm the diagnosis of the disease or reflect an old infection [30]. In contrast, results with the K-K technique are approximate when *S. mansoni* eggs are in small number in the stool sample. As well as in previous studies [15, 24], we noted that there was no association between the prevalence of *S. mansoni* infection and gender. One reason of this finding might be frequency of the visits at the water source that was identical for all PSAC.

Based on the K-K method, *S. mansoni* infection prevalence was significantly higher in PSAC aged 2–6 years. Similar findings from Tanzania [15] also demonstrated that schistosomiasis prevalence increased as PSAC get older. Indeed, children belonging to this age group were most active and autonomous with regard to their parents. However, in Ethiopia [31], a nonstatistically significant relationship was reported. A plausible explanation might be the behaviour of children of this age category.

Using POC-CCA, the overall prevalence for *S. mansoni* infection was 62.86% within 1-<2 years (64.76%) versus 62.04% in 2–6 years. This finding brings up an issue concerning *S. mansoni* infection diagnosed with POC-CCA testing in lactating infants. Presumably, POC-CCA testing in PSAC aged 1-<2 was able to screen antibodies coming from the mother through breastfeeding [32, 33].

In Blapleu, there were two mean risk factors associated with *S. mansoni* infection, namely, children aged 2–6 years and exposure to water source points. Household tasks such as laundry were mainly carried out on various water sources within the community.

The mothers/legal guardians typically visited these water sources together with family children, including PSAC aged 2 to 6 years in order to help them with laundry. Children took this opportunity for bathing in the water source. A recent study reported similar findings except that mothers were the only ones involved in doing the laundry [34]. Several studies revealed other schistosomiasis risk factors such as fishing and swimming as activities practiced by PSAC in an analogous setting [17, 35].

## 5. Conclusion

This study revealed a high prevalence of *S. mansoni* infection among PSAC in Blapleu community by using the POC-CAC diagnostic method and moderate based on the K-K technique. This study also showed that 2–6-year-old PSAC who had frequent contact with water sources in this locality had a higher risk of *S. mansoni* infection. In addition, reported cases of *S. mansoni* infection were mainly distributed in a concentric pattern close to the water source. Schistosomiasis control effort in such localities should include information, education, and communication (IEC) and water, sanitation, and hygiene (WASH). In particular, it appears crucial that mass drug administration (MDA) for the control of schistosomiasis also takes into account the PSAC in this area. This explains the interest in strengthening research for a paediatric praziquantel formulation.

## Figures and Tables

**Table 1:** Sociodemographic characteristics of 1–6-year children in Blapleu, Biankouma District, Western Côte d'Ivoire.

Characteristics	1-<2 years, *n* (%)	2–6 years, *n* (%)	Added values, *n* (%)
*Sex*			
Male	63 (60.00)	130 (53.06)	193 (55.14)
Female	42 (40.00)	115 (46.94)	157 (44.86)

*Mother's education*			
Unschooled	84 (80.00)	184 (75.08)	268 (76.57)
Primary school	18 (17.14)	39 (15.93)	57 (16.29)
College	03 (02.86)	22 (08.99)	25 (07.14)

*Household type*			
Traditional	91 (86.67)	226 (92.25)	317 (90.29)
Modern	14 (13.33)	19 (07.75)	33 (09.71)

*Household size*			
[3–6]	60 (57.14)	175 (71.43)	235 (67.15)
[7-8]	25 (23.81)	38 (15.51)	63 (18.00)
[9-10]	12 (01.43)	23 (09,39)	35 (10.00)
[11-12]	08 (07.62)	09 (03.67)	17 (04.85)

*Household number rooms*			
[1–3]	70 (66.67)	191 (77.96)	261 (74.57)
[4–7]	35 (33.33)	54 (22.04)	89 (25.43)
*Children defecation site*			
Latrine	04 (03.81)	88 (35.92)	92 (19.86)
Open air	31 (29.52)	95 (38.78)	126 (34.15)
Basin/container	70 (66.67)	62 (25.30)	132 (45.99)

*Household water*			
Domestic boring	100 (95.25)	177 (72.26)	277 (83,75)
Paid water pump	02 (01.90)	23 (09,39)	25 (05.65)
Free water pump	01 (00.95)	14 (05,70)	15 (03.33)
Backmatter	02 (01.90)	31 (12.65)	33 (07.27)

*Ethnic group*			
Aboriginal	34 (32.38)	121 (49.39)	154 (44.00)
Allochtone	71 (67.62)	125 (50.61)	196 (56.00)

*Religion*			
Christian	18 (17.14)	65 (26.52)	83 (23.71)
Muslim	57 (54.29)	95 (38.78)	152 (43.43)
Buddhist	00 (00.00)	02 (00.82)	02 (00.57)
No religion	30 (28.57)	83 (33.88)	113 (32.29)

**Table 2:** Prevalences of *S. mansoni* infection according to the diagnostic test, the age, and the gender in 350 PSAC in Blapleu, Biankouma District, Western Côte d'Ivoire.

		*N* examined	*n* infected (%) prevalence	95% confidence interval	*P* value
*POC-CCA*		350	220 (62.86)		
Gender	Male	193	120 (62.18)	55.27–69.08	0.77
	Female	157	100 (63.69)	56.09–71.30	
Age group	1-<2 years	105	68 (64.76)	55.47–74.05	0.63
	2–6 years	245	152 (62.04)	55.92–68.16	

*Kato-Katz*		220	110 (31.43)		
Gender	Male	120	63 (32.64)	43.43–61.56	0.42
	Female	100	47 (29.94)	37.05–56.95	
Age group	1-<2 years	68	11 (10.48)	7.2–25.15	<0.001
	2–6 years	152	99 (40.41)	57.47–72.79	

**Table 3:** Use of water contact points by the 350 PSAC and their mothers in Blapleu, Biankouma District, Western Côte d'Ivoire.

Characteristics	1-<2 years, *n* (%)	2–6 years, *n* (%)	Added values, *n* (%)
*Go to the water contact point*			
Yes	53 (50.48)	119 (48.57)	172 (49.14)
No	52 (49.52)	126 (51.43)	178 (50.86)

*Purpose for visiting the water contact point*			
Helping mother with housework	52 (98.11)	96 (80.67)	148 (89.39)
Friendly bathing	1 (01.89)	23 (19.33)	24 (10.61)

*Children's activities on water points*			
Laundry/bathing	39 (75)	116 (97.48)	155 (86.24)
Carried on mother's back	13 (25)	3 (02.52)	16 (13.76)

*Mother's activities at the water point*			
Laundry	49 (92.45)	88 (75.21)	97 (83.33)
Supplying water	4 (07.55)	26 (22.23)	30 (14.89)
Rice fields	0	5 (02.56)	5 (01.28)

*Mother and water point contact frequency*			
Daily	04 (07.55)	9 (07.56)	13 (07.00)
Weekly	38 (71.70)	99 (83.20)	137 (78.00)
Twice a week	11 (20.75)	11 (09.24)	22 (15.00)

## Data Availability

The data used to support the findings of this study are available from the corresponding author upon request.
